# Expanding the role of Mohs surgery: Management of cutaneous metastases in 3 cases

**DOI:** 10.1016/j.jdcr.2025.12.001

**Published:** 2025-12-10

**Authors:** Alexander M. Hammond, Navid Farahbakhsh, Vladimir Vincek, Sailesh Konda

**Affiliations:** a Department of Dermatology, University of Florida College of Medicine, Gainesville, Florida; b Division of Plastic Surgery, Department of Surgery, University of Florida College of Medicine, Gainesville, Florida

**Keywords:** cutaneous metastases, Mohs micrographic surgery (MMS), palliative therapy

## Introduction

The use of Mohs micrographic surgery (MMS) for the palliative treatment of cutaneous metastatic lesions remains underreported in the literature.^1^^,^^2^ Cutaneous metastases can significantly impact a patient’s quality of life, yet treatment options are often limited.^3^ Herein, we describe 3 patients with symptomatic metastatic cutaneous lesions who were treated with MMS.

## Case report

### Case 1

**Fig 1:**
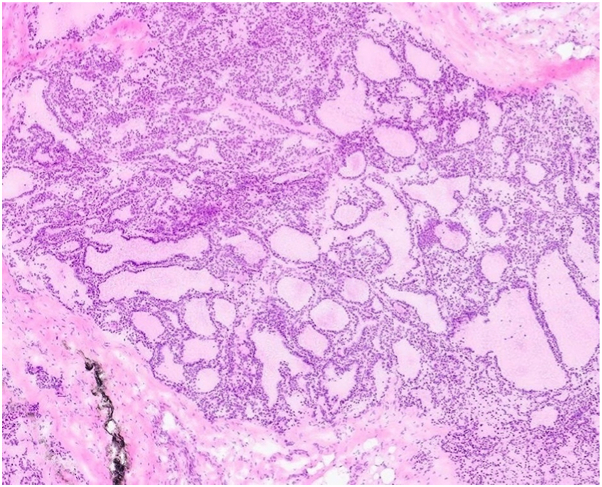
Metastatic follicular thyroid carcinoma with thyroid follicles of variable size and minimal cytological atypia; magnification 200×, hematoxylin and eosin (H&E) stain.

**Fig 2:**
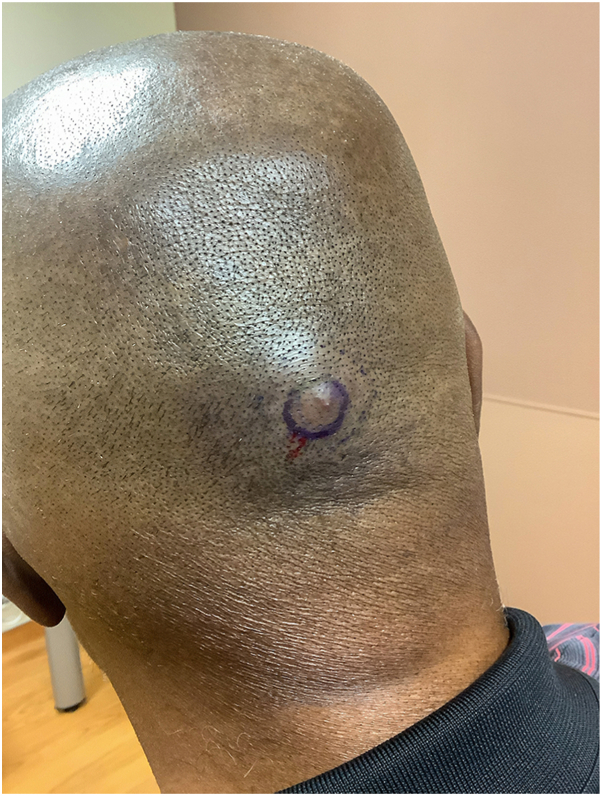
Mid-occipital scalp with 1.5 × 1.3 cm tender, subcutaneous nodule.

A male patient with a history of papillary thyroid carcinoma, status post-thyroidectomy in September 2018, with known metastases to the spine, liver, and lungs treated with radiation, initially presented to dermatology for evaluation of a painful lesion on the left lateral vertex scalp. A biopsy confirmed metastatic thyroid carcinoma (Fig 1), and the patient was scheduled for MMS. At the time of surgery, a second suspicious lesion was noted on the mid-occipital scalp (Fig 2). Intraoperative frozen section biopsy confirmed this lesion was also consistent with metastatic thyroid carcinoma. MMS was performed for both lesions on the left lateral vertex scalp and the mid-occipital scalp on the same day. The tumors cleared in 2 and 3 stages, respectively.

### Case 2

**Fig 3:**
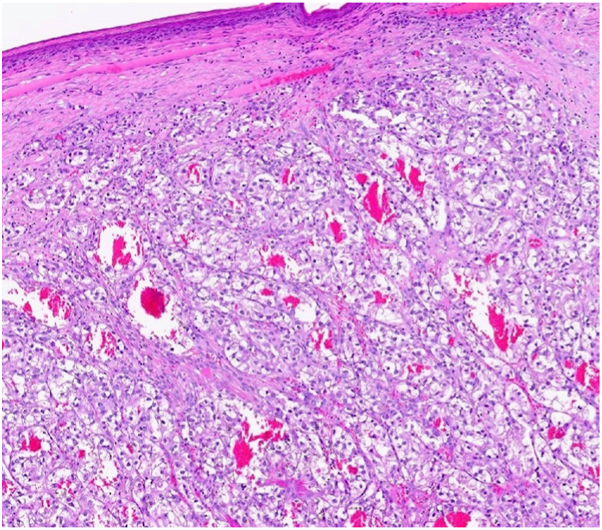
Metastatic renal cell adenocarcinoma, clear cell type, made of nests of irregular clear cells surrounded with prominent vessels, and inconspicuous stroma; magnification 200×, H&E stain. *H&E*, Hematoxylin and eosin.

A male with metastatic renal cell carcinoma undergoing immunotherapy with nivolumab and ipilimumab presented with a painful, friable, and disfiguring lesion on the right upper forehead. Biopsy confirmed cutaneous metastasis from renal cell carcinoma (Fig 3). After discussing treatment options, the patient elected to proceed with MMS. The tumor was successfully cleared in a single stage using frozen sections.

### Case 3

**Fig 4:**
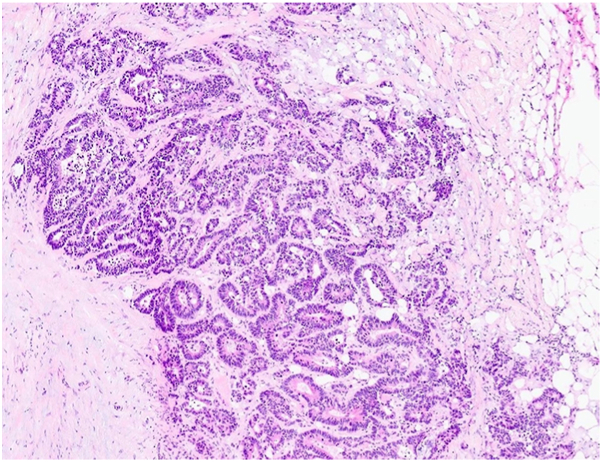
Metastatic, well differentiated colon adenocarcinoma with cribriform glands and focal necrotic debris; magnification 200×, H&E stain. *H&E*, Hematoxylin and eosin.

**Fig 5:**
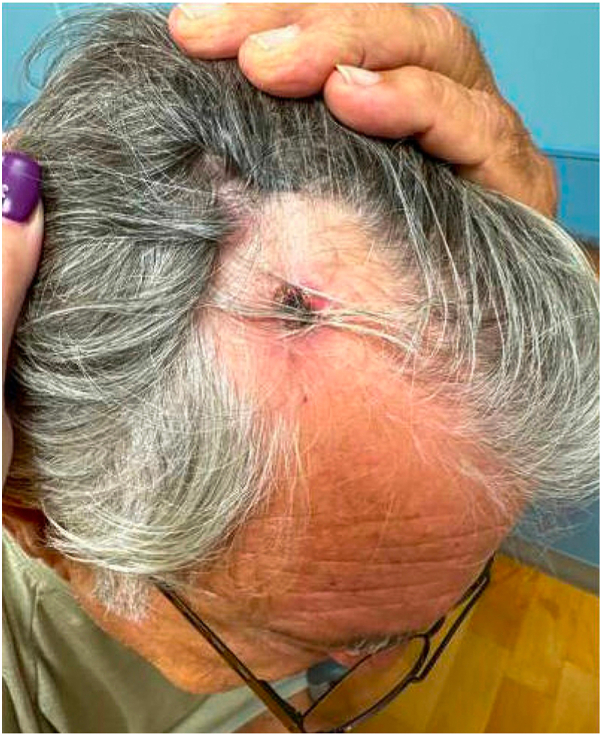
Right superior frontal scalp with 1.4 × 1.2 cm pink, eroded, crusted plaque.

Our third case features a male with metastatic colorectal adenocarcinoma (Fig 4) who had failed prior chemotherapy and radiation. He was referred for surgical management of a painful, cosmetically bothersome metastatic tumor on the right superior frontal scalp that recurred following radiation therapy (Fig 5). Given the patient's desire for symptom relief and improved appearance, MMS was performed. The tumor was successfully excised after 3 stages using frozen sections.

## Discussion

Cutaneous metastasis of internal malignancies is rare and generally associated with a poor prognosis.^1^ These metastases from solid tumors most commonly occur through hematogenous or lymphatic spread.^4^ Clinically, patients typically present with skin-colored, painless, and firm nodules. The location of cutaneous metastases often correlates with the site of the primary tumor. For instance, breast, lung, and kidney cancers most frequently metastasize to the scalp, whereas colon cancer has a predilection for the anterior thorax.^4^ A high index of suspicion is warranted in patients with a known history of malignancy; however, in some cases, cutaneous metastasis may be the initial manifestation of an undiagnosed internal cancer.^5^

Biopsy of the presenting lesion is essential to establish the diagnosis and guide appropriate management. When multiple lesions are present, each should be biopsied to confirm that all suspected sites represent cutaneous metastases. In 1 prior case, a lesion presumed to be a basal cell carcinoma was referred to a Mohs surgeon; however, a biopsy on the day of surgery for permanent sections revealed it to be a metastatic lung adenocarcinoma.^5^

Typically, treatment is directed at the primary tumor, as the presence of cutaneous metastasis signifies advanced disease.^4^ While antineoplastic therapies are the mainstay for managing the primary malignancy, they often have limited effectiveness in addressing cutaneous lesions.^3^ Skin-directed treatment options for cutaneous metastases remain limited. A prior meta-analysis evaluated 5 skin-directed therapies, including electrochemotherapy, photodynamic therapy, radiotherapy, intralesional therapy, and topical therapy. These skin-directed therapies resulted in a complete response rate of 35.5%, an objective response rate of 60.2%, and a recurrence rate of 9.2%.^3^

MMS offers real-time, complete histologic margin assessment, a distinct advantage over conventional wide local excision. This often eliminates the need for serial treatments with other skin-directed therapies that typically require multiple sessions over weeks to months to achieve similar control. While MMS is not curative for these lesions, it allows for complete excision of the local tumor at the surgical site, which can significantly improve symptoms such as pain or bleeding and enhance cosmetic outcomes. In contrast, other treatment modalities—such as topical therapies, radiation, or systemic approaches—typically rely on clinical regression or imaging findings to assess treatment response, without direct histologic confirmation of local clearance. This can be particularly limiting in metastatic disease, where residual microscopic tumor may be clinically occult yet have important consequences for local control and quality of life. Hematoxylin and eosin frozen sections are generally sufficient for intraoperative histologic evaluation. The use of immunohistochemical stains (i.e. CD10 for renal cell carcinoma or thyroid transcription factor-1 for thyroid carcinoma) may provide clarity in poorly differentiated tumors or in tissue with dense inflammation.

The 3 patients described in this paper underwent MMS for symptomatic and cosmetically bothersome metastatic lesions. Biopsies were performed prior to treatment to confirm the diagnosis in each case. One patient had failed previous treatment with radiation therapy. All 3 patients successfully completed MMS without complications. The patient with metastatic thyroid carcinoma remains free of local tumor recurrence 7-months postoperatively. Unfortunately, the individuals with metastatic renal cell carcinoma and metastatic colorectal adenocarcinoma later died from progression of their underlying disease, despite the lack of local tumor recurrence, highlighting the poor overall prognosis associated with cutaneous metastases.

Although not a curative approach for metastatic disease, MMS can serve a valuable palliative role in patients presenting with symptomatic lesions, cosmetic disfigurement, or functional impairment.

## Conflicts of interest

None disclosed.
